# A Rare Aural Complication of Gonorrhea

**Published:** 1893-02

**Authors:** 


A RARE AURAL COMPLICATION OF GONORRHEA.—Fischel (Prager Medicin, Whochenschr, No. 11, 1891), reports the following case: About the twenty-second day of the gonorrheal attack, the patient, age twenty-four, was taken with chill, and developed left-sided epididymitis. Two days later appeared a very severe headache and troublesome tinitus. The following day he became totally deaf in both ears. M. Habermann, who examined the patient with the writer, confirmed the diagnosis. The ear trouble was due to gonorrheal infection. The revulsion and diuretic treatment materially improved the hearing in a few days.—*Brooklyn Medical Journal.*

